# Peptide-based vaccine successfully induces protective immunity against canine visceral leishmaniasis

**DOI:** 10.1038/s41541-019-0144-2

**Published:** 2019-11-29

**Authors:** Elodie Petitdidier, Julie Pagniez, Joana Pissarra, Philippe Holzmuller, Gérard Papierok, Philippe Vincendeau, Jean-Loup Lemesre, Rachel Bras-Gonçalves

**Affiliations:** 1 UMR INTERTRYP, University of Montpellier, IRD, CIRAD, Montpellier, France; 2 CIRAD, UMR ASTRE, F-34398, Montpellier, France; 3 ASTRE, CIRAD, INRA, University of Montpellier (I-MUSE), Montpellier, France; 4 VIRBAC Animal Health, Carros, France; 5 Laboratoire de Parasitologie, CHU Bordeaux, IRD UMR INTERTRYP, University of Bordeaux, Bordeaux, France

**Keywords:** Vaccines, Peptide vaccines, Parasitic infection

## Abstract

Dogs are the main reservoir of zoonotic visceral leishmaniasis. Vaccination is a promising approach to help control leishmaniasis and to interrupt transmission of the *Leishmania* parasite. The promastigote surface antigen (PSA) is a highly immunogenic component of *Leishmania* excretory/secretory products. A vaccine based on three peptides derived from the carboxy-terminal part of *Leishmania amazonensis* PSA and conserved among *Leishmania* species, formulated with QA-21 as adjuvant, was tested on naive Beagle dogs in a preclinical trial. Four months after the full course of vaccination, dogs were experimentally infected with *Leishmania infantum* promastigotes. Immunization of dogs with peptide-based vaccine conferred immunity against experimental infection with *L. infantum*. Evidence for macrophage nitric oxide production and anti-leishmanial activity associated with IFN-γ production by lymphocytes was only found in the vaccinated group. An increase in specific IgG2 antibodies was also measured in vaccinated dogs from 2 months after immunization. Additionally, after challenge with *L. infantum*, the parasite burden was significantly lower in vaccinated dogs than in the control group. These data strongly suggest that this peptide-based vaccine candidate generated cross-protection against zoonotic leishmaniasis by inducing a Th1-type immune response associated with production of specific IgG2 antibodies. This preclinical trial including a peptide-based vaccine against leishmaniasis clearly demonstrates effective protection in a natural host. This approach deserves further investigation to enhance the immunogenicity of the peptides and to consider the possible engineering of a vaccine targeting several *Leishmania* species.

## Canine Leishmaniasis: peptide-based vaccines for protection

Leishmaniasis, caused by the protozoan parasite *Leishmania*, can present in different forms depending on the infecting species. Visceral leishmaniasis is associated with migration of the parasite, in this case *Leishmania infantum*, to various organs and can infect both humans and canids. Here Rachel Bras-Gonçalves and colleagues test a *Leishmania* vaccine for dogs as they are the main reservoir for this zoonotic disease. The vaccine is based on the abundant immunogenic component of *Leishmania* excretory/secretory product, promastigote surface antigen (PSA); specifically, three peptides from the carboxyl-terminal of PSA, which is conserved in *Leishmania* species. Uninfected Beagle dogs were immunized with QA-21 as an adjuvant, and no local or systemic adverse reactions were observed. Four months later after three doses of the vaccine, dogs were infected with *L. infantum* promastigotes. Vaccination provided immunity with reduced parasite burden and this was associated with macrophage anti-leishmanial activity, increased IFN-y and nitric oxide production and increased Leishmania-specific IgG2 antibodies.

## Introduction

*Leishmania* infections are vector-borne neglected diseases caused by *Leishmania spp*. protozoan parasites transmitted by phlebotomine sand flies. Currently, they are responsible for the second-highest number of deaths due to parasitic infections worldwide, and are overwhelmingly associated with poverty.^1,2^

Zoonotic visceral leishmaniasis (ZVL) affects both humans and canids and is caused by *Leishmania infantum/Leishmania chagasi* in the Mediterranean region, several Middle Eastern, African and Asian countries, in South and Central America and probably in southern US.^3,4^ Wild and domestic canids are known to be the main reservoir of parasites, and to continuously supply the transmission cycle of *L. infantum* in the old world and *L. chagasi* in the new world. Canine visceral leishmaniasis (CVL) is a severe disease characterized by chronic evolution of viscerocutaneous signs, which is of great importance in Europe for both public health and in veterinary medicine. At least 2.5 million dogs are probably infected and severely affected dogs do not survive.^5^ Both symptomatically and asymptomatically infected dogs can be considered as a reservoir of the parasite involved in the transmission cycle of *L. infantum* in dogs and humans.^6^ In ZVL-endemic regions of the Mediterranean and Latin America, a high prevalence of canine infection is associated with a high risk of human disease.^7^

Prevention of CVL requires reducing transmission of the parasite, including treatment of dogs based on chemotherapy, and reducing the population of vectors. Applying insecticides may have a transitory effect but is typically unsustainable in the long term for several technical and economic reasons as well as for the protection of the environment. Although substantial progress has been made in current drug treatments, this approach cannot be used to treat asymptomatic infected dogs because they are not diagnosed and are always at risk of developing leishmaniasis.^8^ With a view to long-term and cost-effective protection of dogs and humans against leishmaniasis, preventive vaccination is a very promising approach and hopefully could also be used to interrupt the transmission of *Leishmania* and eliminate leishmaniasis.^6^

A major limitation in the field of leishmaniasis vaccines concerns the difficulty of finding an animal model that reproduces the aspects of natural disease and the immune responses required for efficacy. Past experiments have underlined the danger of extrapolating results from experimental animal models such as rodents to human or dog diseases.^9^ Selection of vaccine candidates is challenging because of the large number of antigens to be evaluated with different levels of effectiveness depending on their formulation and on the animal model used.^10^ Preclinical research in rodent models has provided evidence for the efficacy of several categories of *Leishmania* antigens including whole parasites, cell purified fractions, parasite protein components or subunits, single or multiple chimeric recombinant proteins, plasmid DNA and viral particles encoding parasite virulence factors.^10–13^ Despite the successful protection conferred by the many vaccine candidates in rodent models (mouse and hamster), only two prophylactic vaccine candidates against human leishmaniasis are now in clinical trials (ClinicalTrial.gov identifiers NCT01011309 and NCT01751048).

Dog is an appropriate model to objectively evaluate the effectiveness of a vaccine targeted for ZVL. A small number of vaccine candidates have been tested in dogs^14^ and four vaccines have obtained a commercial license against CVL: (i) Leishmune^®^ in Brazil, a semi-purified fucose–mannose ligand antigen (FML) adjuvanted with Quil-A^®^.^15^ However, the Leishmune^®^ license has been suspended since 2014 as the vaccine did not fulfil the phase III requirements in terms of vaccine efficacy;^16^ (ii) Leish-Tec^®^, the only vaccine currently sold in Brazil, which contains a recombinant protein A2 adjuvanted with saponin;^17^ (iii) CaniLeish^®^ in Europe, composed of *L. infantum* excreted/secreted products (*Li*ESAp) and adjuvanted with QA-21^18–20^ and finally, (iv) LetiFend^®^, which contains Protein Q as active ingredient, has received marketing authorization by the European Commission.^21^

Using *Leishmania* excreted/secreted products, we previously showed that soluble promastigote surface antigens (PSA) were characterized as immunodominant excreted/secreted components of *Leishmania amazonensis* and *L. infantum*.^22^ We recently showed that, 6 months after infection with *L. infantum*, 78.8% of naive vaccinated dogs with recombinant PSA (rPSA, *La*PSA-38S) of *L. amazonensis* and 80% of vaccinated dogs with its carboxy-terminal part (Cter-rPSA), both combined with QA-21 as adjuvant, were protected against experimental infection.^23^ This cross-protection was associated with hallmarks of a dominant Th1-type immune response. We also clearly demonstrated in *L. infantum* and *L. major*-protected humans (healed individuals), that a dominant Th1-type response associated with a cytotoxic response was induced in vitro by rPSA.^24^ The PSA protein is highly abundant in the *Leishmania* secretome, further supporting its use as a vaccine antigen candidate.^22^ However, the low yield of rPSA production precludes its use as second-generation dog or human vaccine.

The aim of the present study was to evaluate a peptide-based vaccine candidate made of immunodominant peptides, selected and derived from the abundant *Leishmania* rPSA. Indeed, protein abundance is a key feature for antigen selection, as abundant proteins are more likely to be processed and presented by major histocompatibility complex (MHC) molecules to T lymphocytes, and protein abundance correlates with peptide immunodominance.^25^ The results of the present study will help guide research towards peptide-based synthetic vaccines, as these have many advantages in terms of safety, efficacy and cross-protection, with reproducible, cost-effective and large-scale production, to fight zoonotic parasitic diseases.

## Results

### Local and general reactions upon vaccination and clinical follow-up post-infection

Fifteen naive Beagle dogs were enroled in this study and randomized into two experimental groups: a control group with five dogs and a vaccinated group composed of ten dogs. No local and systemic adverse reactions were observed in any of the dogs immunized with the peptide-based vaccine candidate or peptide solvent alone. All the animals maintained constant body condition and body weight throughout the study. No hyperthermia was induced. The overall tolerance of the peptide-based vaccine appeared to be satisfactory in all dogs. No obvious clinical sign of leishmaniasis was observed in any of the dogs at any time point during the study.

### Parasitological evolution of dogs

**Fig. 1:**
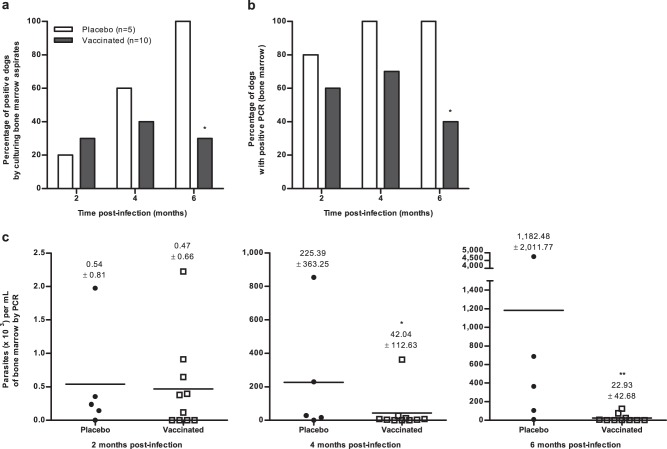
Post-challenge parasitological evaluation of placebo control and vaccinated dogs. **a** The presence of live *Leishmania* parasites highlighted by NNN sub-culture analysis of bone marrow aspirates isolated from dogs in the placebo (*n* = 5) and vaccinated (*n* = 10) groups at 2, 4 and 6 months post-infection. A sample was considered positive when *Leishmania* parasites were detected during seeding or sub-culture analysis. **b** The presence of *Leishmania* DNA and **c** the parasite load in bone marrow aspirates of dogs of each group were assessed by quantitative PCR. Data are expressed as the percentage of positive dogs (**a**, **b**) and the mean number of parasites per millilitre of bone marrow aspirate (**c**) at different times post-infection (2, 4 and 6 months). Dots represent individual animals and lines represent means. Asterisks (*) indicate significant mean differences (*p < 0.05, **p < 0.01) measured by Fisher’s Exact test (**a** and **b**) or Mann–Whitney–Wilcoxon test (**c**).

Four months after the full course of vaccination (three doses), all dogs were experimentally infected by intravenous injection of 10^8^ infective promastigotes of *L. infantum*. Figure 1 shows the percentages of actively infected positive dogs per group found by sub-culturing bone marrow aspirates (Fig. 1a) and by detecting parasite DNA using quantitative polymerase chain reaction (q-PCR) (Fig. 1b) at 2, 4 and 6 months post-challenge (PC). Results of parasite cultures were positive in the control group (*n* = 5), in one (20%), three (60%) and five (100%) dogs at 2, 4 and 6 months, respectively. By contrast, results of parasite cultures in the vaccinated group (*n* = 10) were positive in three (30%), four (40%) and three (30%) animals at the same time points. In detail, from the three infected dogs at 2 months PC, one of these remained positive at 4 and 6 months PC, whereas the two other dogs became negative at these time points. Three new dogs were found culture positive at 4 months PC, for a total of four infected dogs. Two of these four dogs became negative at 6 months PC, the other two dogs remained positive, and one new dog was found culture positive. By the end of the experiment, three animals out of ten were positive 6 months PC, revealing again a significant difference in the percentage of actively infected dogs between the two groups (100% versus 30%, *p* = 0.019). All the control dogs were found to be PCR-positive at 4 and 6 months PC as shown in Fig. 1b. Sixty percent of the vaccinated dogs were PCR-positive at 2 months PC and 70% at 4 months PC, versus only 40% at 6 months PC, i.e. a significant difference from the control group (*p* = 0.042). Group data for bone marrow parasite loads are presented in Fig. 1c. Mean values were significantly lower in the group of vaccinated dogs than in the control group at 4 and 6 months post-infection (*p* = 0.049 and *p* *=* 0.005, respectively).

### Specific IgG2 antibody responses to *Leishmania* antigens

**Fig. 2:**
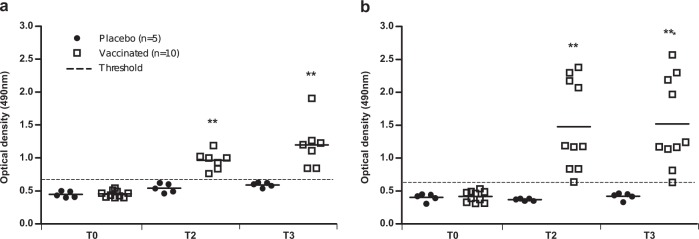
Vaccine-specific serological responses detected by ELISA. **a** Changes in levels of anti-*Li*ESAp and **b** anti-E34PC-specific IgG2 antibodies in serum samples isolated from dogs in each group before immunization (T0) and two (T2) and three (T3) months after the third dose of vaccine. Positivity threshold values were calculated using the following formula: mean OD in sera from all dogs at T0 + 3 standard deviations. Dots represent individual animals and lines represent means. Asterisks (*) indicate significant mean differences (**p < 0.01, ***p < 0.001) measured with the Mann–Whitney–Wilcoxon test.

Specific IgG2 antibody responses against *L. infantum* promastigote excreted/secreted antigens (*Li*ESAp) and E34PC peptide were measured by enzyme-linked immunosorbent assay (ELISA) in all serum samples (Fig. 2). Before immunization (T0), all the dogs in both groups showed a baseline with low levels of specific IgG2 antibodies. A similar low specific IgG2 response was observed in dogs in the control group two (T2) and three (T3) months post-immunization. By contrast, dogs vaccinated with peptides/QA-21 showed significantly higher levels of anti-*Li*ESAp (*p* = 0.001 and *p* = 0.003, Fig. 2a) and anti-E34PC (*p* = 0.001 and *p* = 0.001, Fig. 2b) antibodies at T2 and T3 after immunization, respectively. Overall, our data show a clear post-vaccination peptide-specific IgG2 response in vaccinated dogs.

### Development of macrophage anti-leishmanial activity by vaccination

**Fig. 3:**
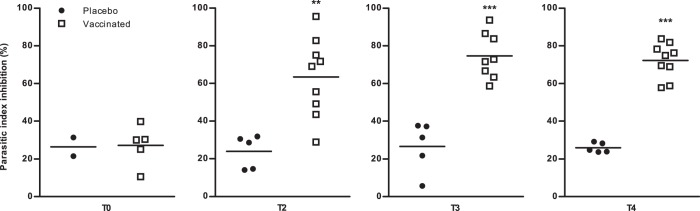
In vitro anti-leishmanial activity of canine monocyte-derived macrophages in non-immune and immune dogs. Canine monocyte-derived macrophages were cultured for 5 days, then infected and exposed to autologous lymphocytes. Their anti-leishmanial activity was expressed as the percentage inhibition of parasitic index and was evaluated before immunization (T0, placebo *n* = 2, vaccinated *n* = 5) and two (T2, placebo *n* = 5, vaccinated *n* = 9), three (T3, placebo *n* = 5, vaccinated *n* = 8) and four (T4, placebo *n* = 5, vaccinated *n* = 9) months after the third dose of vaccine. Dots represent individual animals and lines represent means. Asterisks (*) indicate significant mean differences (**p < 0.01, ***p < 0.001) measured with the Mann–Whitney–Wilcoxon test.

Anti-leishmanial activity is expressed as a percentage of parasite index inhibition (Fig. 3). Before immunization (T0), the assay revealed no significant anti-leishmanial activity in any of the dogs in either the control group or the vaccinated group. By contrast, statistical differences were found between the vaccinated and control groups after the full administration of the vaccine. Indeed, two (T2), three (T3) and four (T4) months post-vaccination, evidence was found for higher macrophage anti-leishmanial activity after 72 h exposure of in vitro infected macrophages to autologous lymphocytes in dogs vaccinated with peptides/QA-21, as demonstrated by a significant parasitic index inhibition: 63%, 75% and 68%, compared to the control group: 24%, 27% and 26% (*p* *=* 0.002, *p* *=* 0.001 and *p* *=* 0.001), respectively.

### Production of IFN-γ cytokine and derivative of nitric oxide

**Fig. 4:**
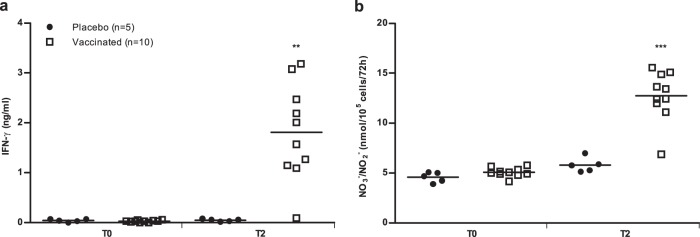
IFN-γ and NO derivative (NO_3_^−^/NO_2_^−^) production by canine macrophages co-cultured for 72 h with autologous lymphocytes from dogs in control and vaccinated groups. **a** IFN-γ levels were determined by a two-site sandwich ELISA in cell culture supernatants. **b** NO_3_^−^/NO_2_^−^ accumulation in the same samples was used as an indicator of NO production by activated macrophages. IFN-γ and NO derivative productions were determined before (T0) and two months (T2) after the third dose of vaccine. Dots represent individual animals and lines represent means. Asterisks (*) indicate significant mean differences (**p < 0.01, ***p < 0.001) measured using a Mann–Whitney–Wilcoxon test.

As shown in Fig. 4a, supernatants of infected macrophages co-cultured with autologous lymphocytes from all the dogs in the control group expressed low IFN-γ levels (about 0.05 ng/mL), prior to immunization (T0) and two months after the third injection (T2). By contrast, at T2, the IFN-γ level was significantly higher in co-cultures of pre-infected macrophages upon activation with autologous lymphocytes from peptide-vaccinated dogs compared to the control group (1.81 ± 0.96 ng/mL, *p* *=* 0.0013).

At the start of the experiment, infected macrophages from all dogs produced small quantities of nitric oxide (NO) derivative (4.91 ± 0.52 nmol/10^5^ cells/72 h, Fig. 4b). At T2, levels of NO derivative produced by infected macrophages from vaccinated dogs were significantly higher (12.75 ± 2.52 nmol/10^5^ cells/72 h), whereas production of NO derivative remained unchanged in cell supernatants from control dogs (5.8 ± 0.73 nmol/10^5^ cells/72 h, *p* = 0.001).

### Conservation of peptide sequences among *Leishmania* species

Among *Leishmania* species, BLAST analysis identified the PSA protein sequences most similar to the *La*PSA-38S protein sequence (GenBank accession number FJ974054; UniprotKB D1GJ50), which are: *Leishmania mexicana* PSA (GenBank accession number FR799565; UniprotKB E9AP03), *Leishmania donovani* PSA (GenBank accession number DQ086111; UniprotKB Q4JI42), *L. infantum* PSA (GenBank accession number FJ974055; UniprotKB D1GJ51), *L. major* PSA (GenBank accession number FR796408; UniprotKB Q4QGL4), *Leishmania tropica* PSA (GenBank accession number AF164027; UniprotKB Q9NDD1) and *Leishmania braziliensis* PSA (GenBank accession number FR798986; UniprotKB A4H6Y8).

**Fig. 5:**
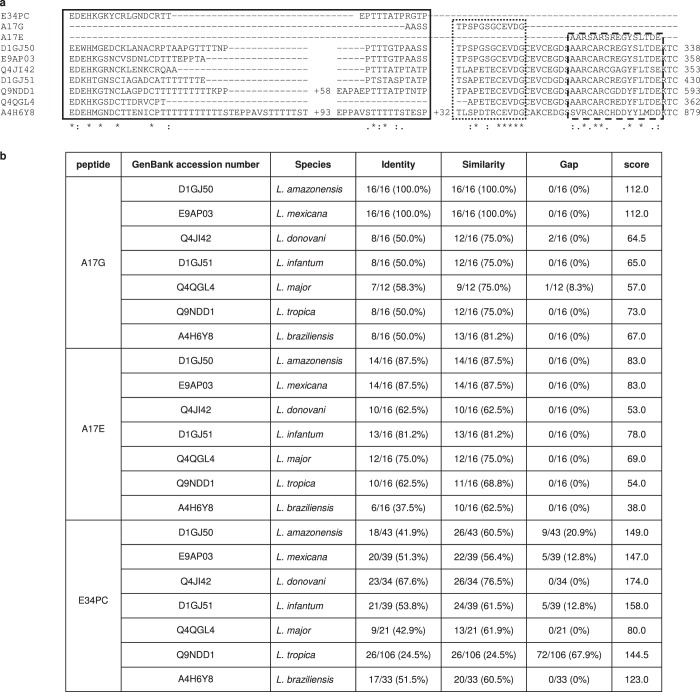
Conservation of peptide sequences among *Leishmania* species. **a** PSA protein sequences from different *Leishmania* species were aligned with A17G, A17E and E34PC peptide sequences using the MUltiple Sequence Comparison by Log-Expectation (MUSCLE) tool. An asterisk (*) indicates positions which have a single, fully conserved residue; a colon (:) indicates conservation between groups of strongly similar properties; a period (.) indicates conservation between groups of weakly similar properties. The full frame, dashed frame and dotted frame represent the E34PC, the A17E and the A17G peptide sequence localizations, respectively. **b** Percentages of identity, similarity and gap between A17G, A17E and E34PC peptide sequences and PSA protein sequences from different *Leishmania* species were calculated on query cover using the EMBOSS Water tool. Percentages were calculated with BLOSUM 30 matrix with the default gap open at 10 and gap extend of 0.5.

Multiple sequence alignment performed with the MUltiple Sequence Comparison by Log-Expectation (MUSCLE) tool revealed that the *La*PSA-38S protein sequence was very similar to PSA proteins from other *Leishmania* species, especially the end amino acid sequences from which our peptide sequences were derived (Fig. 5a).

The EMBOSS Water tool allowed us to show local pairwise sequence alignment of the three peptide sequences A17G, A17E and E34PC and the different PSA protein sequences (Fig. 5b). The A17G and the original A17E peptide sequences were identical to part of the carboxy terminal region of the PSA protein sequence from *L. amazonensis* and *L. mexicana*. However, replacing cysteines by serines in the A17E peptide sequence, to avoid disulphide bridge formation, reduced the identity to 87.5%. The A17E sequence was relatively conserved among PSA protein sequences since it shared at least 60% sequence identity and similarity with them.

E34PC is composed of two distinct parts of consensus sequences, EDEHKGKYCRLGNDCRTT and EPTTTATPRGTPTPAP. These canonical sequences were sometimes separate in some PSA protein sequences but became closer, thanks to link cysteines which form a disulphide bridge. Among *Leishmania* species, the E34PC sequence was partially conserved in the PSA protein sequences. The PSA sequences from *L. donovani* and *L. infantum* were found to be the most closely related with 67.6% identity/76.5% similarity and 53.8% identity/61.5% similarity, respectively.

## Discussion

Wild and domestic dogs are considered to be the main reservoir of *L. infantum/L. chagasi*, responsible for ZVL.^6^ Several drugs are used to cure symptomatic dogs, but infected asymptomatic ones are not included because they are not diagnosed, thus contributing to parasite transmission. In this context, vaccination is a key to interrupting parasite transmission. However, research may be hampered by requirements linked to the development of a vaccine directed against a parasitic and neglected infectious disease. The ideal vaccine must ensure large-scale, reproducible and cost-effective production, and obviously have no or only limited side effects. A peptide-based vaccine would be an effective way to solve these problems. Immunodominant peptides from a *Leishmania* protein of interest were tested as a vaccine, to evaluate their effectiveness against an experimental CVL, by analyzing the induced specific immune response after vaccination and the protection rate after infection of the dogs.

Here, we report that three immunizations with a combination of three synthetic peptides adjuvanted with QA-21, conferred satisfactory cross-protection against an experimental challenge with virulent *L. infantum* promastigotes in dog. Live parasites from bone marrow aspirates, a sign of active infection, were not detected in 70% of vaccinated dogs at 6 months PC, whereas all the dogs in the control group were found to be positive at this time point. The parasite burden of the infected vaccinated dogs was significantly lower than that of dogs in the control group. Humoral and cellular immune responses revealed clear differences between vaccinated and control dogs. The peptide-based vaccine provided protection by inducing a Th1-type cellular immune response: production of IFN-γ and NO derivatives led to intracellular parasite killing. The cellular response was associated with early production of specific anti-E34PC-peptide IgG2 antibodies, and also with the production of specific antibodies targeting the excretory/secretory products of *L. infantum* (*Li*ESAp), which have been correlated with protection.^18,19^ While it has been demonstrated that IgG1 antibodies are associated with susceptibility, disease severity and correlate with a Th2 response, IgG2 antibodies are predominant in naturally resistant or vaccinated dogs and are associated with an appropriate dominant Th1-type immune response.^23,26^

In a previous study, we showed that vaccination with crude *Li*ESAp adjuvanted with muramyl dipeptide (MDP) conferred 100% protection (with a dominant Th1-type response) against an experimental infection with *L. infantum* promastigotes.^18^ Moreover, *Li*ESAp adjuvanted with QA-21 protected dogs exposed to two *L. infantum* transmission seasons in two endemic areas of the Mediterranean basin.^27^ Thanks to these previous studies, which enabled the successful development of the efficient CaniLeish^®^ vaccine,^18–20,26–28^ we identified the secretome as an optimal starting point for the next generation of vaccines against canine leishmaniasis. Based on this observation, in another study, we showed that a rPSA was selectively recognized by sera from *Li*ESAp/QA-21-vaccinated and protected dogs.^22^ More recently, we proved that a vaccine composed of rPSA (*La*PSA-38S, 371 amino acids), or its carboxy-terminal part (*La*PSA-12S, 119 amino acids, representing 32% of the entire rPSA), adjuvanted with 60 µg of QA-21, conferred respectively 78% and 80% of protection against an experimental *L. infantum* infection to vaccinated dogs.^23^ In the present study, we demonstrate that three short peptides (72 amino acids, representing 19.4% of rPSA), derived from this rPSA and formulated with only 20 µg of QA-21, protected 70% (using culture) and 60% (by detecting parasite DNA using q-PCR) of vaccinated dogs against an experimental *L. infantum* infection. These represent a significant percentage considering (i) the immunogenic peptides represent a small fraction of the full recombinant protein tested previously and (ii) the decrease in the adjuvant dose, used in this vaccine formulation which can be optimized in future formulations. Some of the first evidence that a peptide-based vaccine could provide protection against *Leishmania* infection was obtained with experimental mouse models. Two T-cell epitope peptides derived from gp63, one of the main surface glycoproteins of *L. major*, provided significant protection to CBA mice against severe cutaneous lesions.^29^ Many studies on *Leishmania* antigen-derived peptide vaccines are described in literature; for a review, see De Brito et al.^30^ But most studies that describe effective protection against leishmaniasis provided by a peptide vaccine were performed in experimental mouse models. By comparison with similar studies done with commercially available canine vaccines, the efficacy of this peptide-based vaccine is promising. For the Leish-Tec^®^ vaccine (Brazilian vaccine), Beagle dogs were immunized with 100 µg of recombinant protein A2 adjuvanted with 250 µg of saponin, in three injections, and then experimentally infected with 5 × 10^7^ *L. chagasi* promastigotes. Results showed that these immunizations were immunogenic and induced partial protection in dogs (43% by culturing bone marrow aspirates).^17^ Regarding LetiFend^®^ vaccine (one of the European vaccines), Beagle dogs were vaccinated with one injection of 100 µg of recombinant protein Q, without adjuvant, then experimentally infected with 5 × 10^5^ *L. infantum* promastigotes. Parasitological evaluation was performed by culturing lymph node and spleen samples, where 71% and 57% of the dogs were found positive in the vaccinated group, respectively (100% in the control group). Nevertheless, only 14% of the dogs were parasite-DNA positive in eyelid skin samples in the vaccinated group (85% in the control group).^31^

Considering initial vaccination attempts using parasite protein components or subunits and the difficulties involved in producing and purifying recombinant vaccines, the use of selected immunodominant synthetic peptides is a promising alternative. In recent years, peptides have emerged as the best vaccine candidate for human use owing to their simple and cost-efficient production and development process in comparison to traditional vaccines made of whole organisms or large proteins. Many peptide vaccines suitable for human administration are under development, including therapeutic anti-cancer vaccines and vaccines against a number of human infectious diseases.^32–36^ Using synthetic vaccines composed of short immunodominant peptide fragments is an attractive alternative way to elicit targeted immune responses, consequently avoiding allergenic and/or reactogenic sequences.^32,37^ However, peptides can be poorly immunogenic and need to be delivered with additional immune-stimulating agents, such as adjuvants or delivery systems/carriers.^38–40^ The adjuvant is one of the main keys to success. Progress in adjuvant properties and in the role of nanoparticles inducing a Th1-type response has recently been reported.^41^ What is more, chemical changes can also improve peptide vaccine efficacy, binding to MHC molecules and the overall quality of these synthetic peptides. For example, several studies have shown that adding a palmitoyl tail to peptides has advantages including greatly influencing membrane permeability,^42^ enabling the association with MHC class I in dendritic cells^43^ as well as increasing the life span of functional presentation to cytotoxic T cells.^44^

Dogs and humans share a similar type of protective immune response against leishmaniasis, namely a polarized Th1-type response.^45–47^ Results obtained in infected dogs are more reliable and predictive than murine models and can therefore benefit the development of vaccines for humans. Moreover, the development of vaccines able to protect canids from *Leishmania* infection and/or to prevent disease progression is highly desirable not only for the veterinary community but also for the implementation of human leishmaniasis control programs through the reduction of the animal reservoir.^48,49^ A decrease in the incidence of human leishmaniasis after dog vaccination campaigns has been described in Brazil,^50^ highlighting the importance of integrated control programs following a ‘One Health’ approach.

Results obtained in this investigation favour the incorporation of these peptides in a future vaccine approach for dogs and humans. The sequence alignments showed high conservation levels among the A17G and A17E peptide sequences and PSA sequences from *Leishmania* species responsible for the most severe leishmaniases, and partial conservation for the E34PC sequence. Discovering the most conserved epitopes among the antigens mediating protection from different *Leishmania* species will be essential to design a polyvalent vaccine. Despite the fact this study was performed in a short-term survey, it has to be considered as a pilot study. However, future improvements must be performed to relieve control programs, such as the reduction or suppression of boosts. This would make vaccination campaigns logistically more feasible in the areas where leishmaniasis infections occur. Moreover, the addition of other immunodominant peptides should enhance vaccine efficacy. Peptide-based vaccines are safe, their production is simple, reproducible and cost-effective, and they fulfil the necessary field conditions such as stability and storage temperature,^51,52^ which surely provide a great opportunity to interrupt *Leishmania* transmission. The encouraging results we obtained argue for the use of peptides as promising candidates to be included in a new vaccine concept aimed at providing large-scale protection for the target population against leishmaniases.

## Methods

### Vaccine candidate

The peptide sequences were derived from mapped epitopes of the *La*PSA-12S protein, corresponding to the *La*PSA-38S protein carboxy-terminal region (peptide patents WO 2003025012 and WO 2009153458; *La*PSA-38S GenBank accession number: FJ974054 and UniprotKB: D1GJ50; *La*PSA-12S GenBank accession number: FJ974053.1 and UniprotKB: D1GJ49).

For the peptide design, proteasome cleavage analysis of the *La*PSA-12S protein was performed using the NetChop 2.0 Server.^53,54^ The putative cleavage sites predict two 16-mer peptide sequences, named A16G and A16E. The putative antigenicity of these two peptides was then analyzed with VaxiJen v2.0, a prediction server for protective antigens and subunit vaccines,^55^ and both peptides are predicted to be antigenic (score > 0.5). These two peptides were modified as lipopeptides obtained with N-terminal addition of a lysine modified by N^ε^-palmitoylation (K(Pal))—peptides A17G and A17E. In the A17E peptide sequence, the cysteines of the original peptide sequence (AARCARCREGYSLTDE) were replaced by serines (underlined in the sequences) in order to avoid disulphide bridge formation, folding or multimerization of the peptide. Also, an overlapping peptide library was used to discover B-cell epitopes recognized by a monoclonal antibody (mAb F5) directed against excreted/secreted antigens from *L. amazonensis*.^22^ From this analysis performed by Pepscan System company (Lelystad, The Netherlands), a 34-mer peptide has been identified—E34P peptide. The E34P peptide was synthesized as cyclic peptide (and named E34PC peptide) by linking the Cys9 residue to the Cys15 residue of the peptide (underlined in the sequence) with a chemically stable disulphide bond.^56^

Finally, the peptide-based vaccine comprises these three synthetic peptides:

A17G: [K(Pal)-AASSTPSPGSGCEVDG]

A17E: [K(Pal)-AARSARSREGYSLTDE]

E34PC: [EDEHKGKYCRLGNDCRTTEPTTTATPRGTPTPAP].

Each peptide was manufactured under current good manufacturing practice (GMP) conditions by PolyPeptide Group (PolyPeptide Laboratories, Strasbourg, France) as a freeze-dried product. The formulated vaccine consists in 25 µg A17G, 25 µg A17E, 10 µg E34Pc and 20 µg of QA-21 as adjuvant. It is reconstituted in a final volume of 1 mL buffered saline before use.

### Ethics statement

This investigation conformed to the Guide for the Care and Use of Laboratory Animals (NIH Publication No. 85–23, revised 1996). The local Ethical Committee of the National Veterinary School of Lyon (ENVL, France) confirmed that experiments, maintenance and care of dogs complied with guidelines of the European Convention for the Protection of Vertebrate Animals used for Experimental and other Scientific Purposes (CETS no. 123). According to recommendations of this ethic committee for this project (number 136/08), the number of animals and long-term experimentations was kept to the minimum.

### Animals and study design

In order to respect the “three R” rule concerning animal welfare (Replace, Reduce, Refine), sample size calculation was performed with G*Power software, version 3.1.9.2 (University of Kiel, Germany). To compute required sample size, we hypothesized that (i) all dogs from the control group (unvaccinated dogs) should be infected after parasitic challenge (100% of dogs, based on our previous studies), (ii) the vaccine should protect at least 55% of dogs to be consider as effective and (iii) a 2:1 ratio between vaccine and placebo group should be relevant to evaluate protection level. A one-sided test was applied, at an alpha risk of 5% and a power of 80%. Hence, 15 naive Beagle dogs were enroled in this study. Eight males and seven females, between 2 and 4 years old, were selected on clinical and serological criteria in the kennel CEDS (Domaine des Souches, Mezilles, France), a non-endemic *Leishmania* area. The selected dogs were housed at the animal facility of the National Veterinary School of Lyon (ENVL, France) in the preclinical investigation unit throughout the duration of the study under conditions to exclude any possibility of natural *Leishmania* infection. Dogs were housed under the supervision of a veterinarian and had received their routine vaccinations. They were kept in quarantine for a period of 30 days before the study began. A specific code/ID for each dog was used throughout the experiment. Prior to vaccination, blood was collected from all the dogs. Genomic DNA was extracted in order to exclude any infected dog.

Animals were randomized by gender, age and weight into two experimental groups and the study was performed in a double-blind randomized fashion. Dogs of each group received three subcutaneous injections at 28 day intervals of either 1 mL buffered saline (Control group, *n* = 5) or 1 mL formulated vaccine (Vaccinated group, *n* = 10). Four months after the full course of vaccination, all dogs were experimentally infected by intravenous injection of 10^8^ infective promastigotes of *L. infantum* (MHOM/MA/67/ITMAP-263 strain, clone 2). Primary cultures of virulent promastigotes were established from amastigotes isolated from heavily infected mice spleen (BALB/c) and used for the virulent challenge.

### Clinical follow-up and assessment of parasite load

The dogs were monitored after each injection of the vaccine candidate. Local tolerance was investigated by visual examination and any lesions were scored daily over a period of 14 days after each injection. A daily health check, including rectal temperature measurement, was performed. Each dog was also weighed once a week throughout the experiment. The health status of the animals (appetite, physical examination and physical activity) was monitored by veterinarians. After experimental infection, dogs were monitored for parasite establishment and subsequent development of the disease by screening for classical clinical signs, until 6 months after administration of the parasites. Infection of dogs was assessed by collecting bone marrow aspirates (by sternal puncture after anaesthesia) at 2, 4 and 6 months PC. These bone marrow samples were (i) cultured in NNN diphasic medium at 27 °C and checked microscopically for parasite growth weekly and (ii) used to quantify *L. infantum* kinetoplast DNA by real-time q-PCR.

In detail, 0.5 mL of bone marrow samples were cultured in NNN biphasic medium, containing 2 mL of RPMI-20% heat-inactivated foetal calf serum, for 1 week. Subcultures were performed weekly (3 in total) by adding 0.5–1 mL of culture sample in NNN medium containing 3 mL of RPMI-20% FCS. Parasite presence was determined by microscopic observation in an inverted microscope at 400x magnification. A sample was considered as positive when live parasites were observed. For *Leishmania* kinetoplast DNA amplification, DNA of each bone marrow sample was extracted using the QIAamp DNA mini kit (Qiagen) according to the manufacturer’s instructions. The qPCR master mix was composed of forward primer (CTTTTCTGGTCCTCCGGGTAGG, 15 pmol), reverse primer (CCACCCGGCCCTATTTTACACCAA, 15 pmol) and TaqMan probe (FAM-TTTTCGCAGAACGCCCCTACCCGC-TAMRA, 50 pmol). Assays were performed with 1 µL of DNA sample in a final volume of 25 µL. Standard curve was established from *Leishmania* gDNA extracted from 5 × 10^6^ parasites: 1 µL of each serial dilution, ranging from 5 × 10^4^ to 10^−4^ parasites, was introduced into reaction tubes. Comparative quantification was performed using the polymerase gene as house-keeping gene. This gene was amplified with a forward primer (TGTCGCTTGCAGACCAGATG, 200 pmol), reverse primer (GCATCGCAGGTGTGAGCAC, 200 pmol) and TaqMan probe (VIC-CCAGGCTCGAAGTTGTTGCTGCCC-TAMRA, 200 pmol). Reactions were performed on the Stratagene MX4000 Real-Time QPCR System (La Jolla, California, USA) using two-step temperatures (94 °C and 55 °C) cycling over 45 cycles. A sample was considered as positive when the established parasite concentration was superior to 40 parasites per millilitre.

### Detection of specific IgG2 to *Li*ESAp and E34PC peptide by ELISA

Specific IgG2 antibody responses against *L. infantum* promastigote excreted/secreted antigens (*Li*ESAp) and E34PC peptide were measured in the serum samples of all the dogs in both the control and vaccinated groups. These assays were performed prior to immunization and 2 and 3 months after immunization using a standard ELISA procedure.^19,23^ Briefly, sera were added in triplicate at 1/100 dilution in PBS containing 0.05% Tween-20 (PBS-T) to 96-well plates (Nunc MaxiSorp™ Flat bottom 96-well plates) previously coated with *Li*ESAp (0.1 µg per well) or E34PC peptide (0.1 µg per well), and incubated for 1 h at 37 °C. After incubation and three washes in PBS-T, the plates were incubated for 30 min at 37 °C with secondary antibody (horseradish peroxidase-conjugated sheep anti-dog IgG2, 1/5000) (Bethyl Laboratories, Inc., Mongomery, TX, catalogue number A40-121P). After washing in PBS-T, plates were then developed with *o*-phenylenediamine dihydrochloride (OPD) substrate in the dark and absorbance was read at 492 nm. For analysis, a positivity threshold was calculated using the following formula: mean OD in sera collected from all dogs at the starting point (before immunization) + 3 standard deviations. Humoral response was considered positive when the IgG2 titre was above the positivity threshold.

### Assessment of canine monocyte-derived macrophage anti-leishmanial activity

An ex vivo canine macrophage-autologous lymphocyte co-culture system was used to investigate the ability of macrophages to kill intracellular *Leishmania* parasites. Briefly, peripheral blood mononuclear cells (PBMCs) were obtained from heparinized peripheral blood by density centrifugation through Ficoll-Hypaque (GE Healthcare Life Sciences). Canine monocyte-derived macrophages (CM-DM) were isolated from lymphocytes by differential adherence. CM-DM were cultured for 5 days at 37 °C and 5% CO_2_ in RPMI 1640 medium (BioWhittaker, Inc., Walkersville, MD), supplemented with 2 mM glutamine, 10% heat-inactivated FCS, 100 µg/mL streptomycin and 100 IU/mL penicillin. They were then infected with stationary-phase promastigotes of *L. infantum* (MHOM/MA/67/ITMAP-263 strain, clone 2) at a parasite:macrophage ratio of 5:1 for 150 min in 16-well glass culture slides (Nunc™ Lab-Tek™ II Chamber Slide™). After serial washings to remove non-internalized parasites, infected macrophages were incubated alone or in the presence of autologous lymphocytes (previously maintained in culture at 37 °C and 5% CO_2_ in RPMI 1640 medium supplemented with 2 mM glutamine, 10% heat-inactivated FCS, 100 µg/mL streptomycin and 100 IU/mL penicillin) at a lymphocyte:macrophage ratio of 2:1 and incubated for 72 h at 37 °C and 5% CO_2_. Supernatants were then collected for further analyses and macrophages were fixed with methanol and stained with Giemsa. To assess the anti-leishmanial activity, the percentages of infected cells and the number of amastigotes per macrophage were estimated in duplicate experiments by microscopic examination and used to calculate the parasite index (PI) inhibition. Percentage of PI inhibition = 100 − [(mean number of amastigotes per macrophage × percentage of infected macrophages when macrophages were co-cultured with autologous lymphocytes)/(mean number of amastigotes per macrophage × percentage of infected macrophages in non-co-cultured macrophages)] × 100.

### IFN-γ cytokine and NO measurements

Supernatants were collected from macrophages infected 72 h previously and co-cultured with autologous lymphocytes. IFN-γ levels in cell culture supernatants were assessed in triplicate experiments by a quantitative sandwich ELISA according to the manufacturer’s instructions (Canine IFN-γ DuoSet ELISA, R&D Systems, Minneapolis, USA, catalogue number DY781). Nitrate (NO_3_^−^)/nitrite (NO_2_^−^) accumulation in supernatants was measured in triplicate experiments by the Griess reaction as an indicator of NO production by activated macrophages. This colorimetric assay was used following the manufacturer’s instructions (Nitric Oxide (NO_3_^−^/NO_2_^−^) detection kit, Alexis biochemicals, Enzo Life Sciences, France).

### Bioinformatic studies

The A17G, A17E and E34PC peptide sequences were derived from the *La*PSA-38S protein sequence (GenBank accession number: FJ974054;^22^ UniprotKB D1GJ50). PSA protein sequences from the other *Leishmania* species were obtained from Basic Local Alignment Search Tool (BLAST) algorithm (https://blast.ncbi.nlm.nih.gov/). The sequences closest to *La*PSA-38S protein sequence were selected to align with peptide sequences. Putative or hypothetical protein sequences were rejected. The Multiple Sequence Comparison by Log Expectation (MUSCLE) tool (https://www.ebi.ac.uk/Tools/msa/muscle/) was used to align all the selected PSA protein sequences. The pairwise sequence local alignments were obtained using the EMBOSS Water tool (https://www.ebi.ac.uk/Tools/psa/emboss_water/). Percentage identity and similarity were calculated with matrix BLOSUM30 and the default gap open of 10 and gap extend of 0.5.

### Statistical analysis

Data analysis was performed with GraphPad Prism version 5.03 for Windows (GraphPad Software, San Diego, USA). The statistical significance of differences between independent sample groups was determined with the Mann–Whitney–Wilcoxon test and Fisher’s exact test for contingency table analyses. One-sided tests were applied at an alpha risk of 5%. A *p*-value ≤ 0.05 was considered significant.

### Reporting Summary

Further information on research design is available in the Nature Research Reporting Summary linked to this article.

## Supplementary information

Reporting Summary

## Data Availability

The data supporting the findings of the study are available from the corresponding authors upon reasonable request.
